# Late onset oral treatment with tranilast following large myocardial infarction has no beneficial effects on cardiac remodeling and mortality in rats

**DOI:** 10.3892/etm.2014.2003

**Published:** 2014-10-06

**Authors:** STEFAN BETGE, CHRISTIAN KUNZ, HANS FIGULLA, CHRISTIAN JUNG

**Affiliations:** 1 Department of Internal Medicine I, Division of Cardiology, Friedrich Schiller University, Jena, Thuringia 07740, Germany; 2 Department of General, Abdominal and Thoracic Surgery, City Hospital Dessau, Dessau-RoBlau, Saxony-Anhalt 06847, Germany

**Keywords:** ischemic heart failure, antifibrotic, transforming growth factor, fibroblast, collagen content

## Abstract

Tranilast (Tra) reduces intracardiac interstitial fibrosis in the animal models of hypertensive heart failure and diabetic cardiomyopathy by inhibiting cardiac fibroblasts. The present study examined whether Tra has long-term effects on the cardiac remodeling in the remote area of the left ventricle (LV) following myocardial infarction (MI) in the rat. Treatment with Tra (n=40; 150 mg/kg twice daily) or placebo (Plac, n=36) was started at day 28 after induction of a large MI or sham-operation (ShO, n=18) in female Lewis rats. Collagen content was determined using high-performance liquid chromatography. Large MI led to a significant hypertrophy of the two ventricles, a severe dilatation of the LV and a shift of the chamber stiffness variables in the pressure volume curves. The six-month survival rates were Tra, 62.5%; Plac, 75%; and ShO, 100%. No significant difference was identified between Tra and Plac regarding survival rate and collagen content. Treatment with the anti-inflammatory and antifibrotic drug, Tra, started four weeks after the induction of a large MI in the rat, did not attenuate or positively influence remodeling in chronic ischemic heart failure and survival. Further studies are required to explore the effects of Tra on cardiac myocytes post-MI in more detail.

## Introduction

Tranilast was introduced as an anti-atopic agent forty years ago (1). Several studies and clinical reports have described anti-inflammatory and antifibrotic effects in the following years and tranilast was used for the treatment of dermatological disorders, such as scleroderma, cheilitis granulomatosa, granuloma anulare or sarcoidosis (2,3). In cell culture experiments, it has been shown that tranilast inhibits collagen synthesis in fibroblasts, partially as a direct effect on the protein expression and partially via inhibition of the stimulating effect of transforming growth factor β1 (TGF-β1) on the collagen synthesis of fibroblasts (4,5). Tranilast prevented intraperitoneal adhesions in an animal model (6) and it has been used for the prevention of hypertrophic scar development following sternotomy in children (7).

Tranilast inhibited the collagen synthesis and proliferation of vascular smooth muscle cells in cell culture experiments by interacting with the receptors for the platelet-derived growth factor, TGF-β, and angiotensin II (8). These effects could be translated in a significant reduction of the intimal hyperplasia subsequent to treatment with tranilast in different animal models following arterial injury (9), into reduced restenosis rates subsequent to percutaneous coronary angioplasty of *de novo* or restenotic lesions in smaller human studies (10,11), but not in the large Prevention of REStenosis with Tranilast and its Outcomes trial (11,12). In addition, tranilast showed positive effects on intracardiac inflammatory processes positively influencing cardiac remodeling in several animal models (13,14): Tranilast administered once daily for four weeks led to a decrease of the left ventricular hypertrophy and ameliorated the perivascular and interstitial intracardiac fibrosis in spontaneously hypertensive rats. The left ventricular end diastolic pressure or chamber stiffness constants were not affected (13). Similar results were obtained in the animal model of uni-nephrectomized deoxycorticosterone acetate (DOCA)/salt hypertensive rats. Tranilast administered for 28 days attenuated the intracardiac perivascular and interstitial fibrosis, in parallel with an inhibition of TGF-β_1_ expression and a suppression of cardiac mRNA levels of different cytokines (14).

In contrast to these animal models the processes leading to the cardiac remodeling following myocardial infarction (MI) do not involve the entire circumference of the left ventricle (LV). Cardiac remodeling following large MI can be interpreted as a response of the remote non-infarcted section of the LV to changes in ventricular geometry. However, the key features of cardiac remodeling are the same, involving myocyte hypertrophy, apoptosis and interstitial fibrosis. Several data show that the inflammatory processes in the ischemic area, as well as in the remote non-infarcted area of the LV following MI, are involved in the different phases. TGF-β is believed to play a central role in each of these phases. TGF-β promotes extracellular matrix protein expression by fibroblasts and inhibits matrix degradation via several mechanisms (15).

In experimental models of MI the expression of different isoforms of TGF-β was found to be upregulated in different phases post-infarction. TGF-β_1_ and -β_2_ are induced in the early phase, whereas TGF-β_3_ shows delayed and prolonged upregulation (16). The process of cardiac remodeling outside the infarcted area of the LV starts within hours following the infarction and continues to progress over weeks or months (17). An animal study with mice revealed that the blockade of TGF-β-signaling by overexpression of the extracellular domain of the TGF-β type II receptor during the early phase following MI resulted in left ventricular dilatation and increased early mortality. By contrast, blockade of the TGF-β signaling in the later phase following MI prevented the LV dilatation and the reduction of the contractile function, as well as myocyte hypertrophy and interstitial fibrosis (18). Recently, the study by See *et al* (19) reported on early and late administration of tranilast following MI (early, between 24 h and seven days post-MI; late, 7–28 days post-MI). The study revealed that tranilast inhibited myocardial TGFβ_1_ expression, fibrosis in rat post-MI and collagen production in cardiac fibroblasts. However, tranilast intervention from 24 h post-MI exacerbated infarct expansion, delaying the commencement of treatment to seven days post-MI impeded LV remodeling. Therefore, the aim of the present study was to investigate extremely late tranilast administration (starting at day 28) and its effect on cardiac remodeling and the six-month mortality rate in an experimental model of chronic ischemic heart failure following a large MI in the rat.

## Materials and methods

### Animal model and study groups

Studies were performed on 268 female Lewis rats, inbred and raised in the Institute for Animal Experiments of the Friedrich Schiller University (Jena, Germany). The studies were approved by the Ethics Committee of the Friedrich Schiller University. The investigation conforms to the Guide for the Care and Use of Laboratory Animals published by the National Institutes of Health (NIH; publication no. 85-23, revised 1996) and to the German law on the protection of animals.

The study was designed in an intention-to-treat manner. During the randomization process the animals were designated to the different surgical/treatment groups (MI/tranilast, MI/placebo or sham-operation (ShO)/placebo), as well as to the different analysis groups (collagen content via high-performance liquid chromatography (HPLC) and resting pressure-volume-curve/histological studies) prior to surgery. For the induction of an MI, the proximal left anterior descending coronary artery was ligated via left lateral thoracotomy following tracheotomy for controlled ventilation (20). In the sham-operated animals, the suture only was loosely tied. Ribs, muscles and skin were closed in separate layers. The animals were housed for six months, four of each in one polyethylene cage, with a maintained 12 h light/dark cycle. Animals had free access to standard food and water *ad libitum*.

Due to a high mortality rate of 39.3% (97 animals) following ligation of the proximal left anterior descending coronary artery and 4.8% (one animal) following the ShO in the first 48 h after surgery, a total number of 268 animals were randomized and the surgery performed according to the protocol until the intended group sizes of n=75 in each group following MI and n=20 following the ShO were reached. No difference was identified between the groups concerning body weight or age (Table I).

### Drug administration

The administration of the study drug or placebo was performed at days 28–182 post-operation by gavage. The scar formation following MI was completed, so that the treatment with tranilast did not have an effect on the scar formation in the infarcted area of the LV. Tranilast was administered at a daily dose of 300 mg/kg in two doses, dispersed in a solution of Tylose H 300, which served as the placebo as well.

### Preparation

The rats that succumbed during the study period were autopsied and the hearts and lungs were excised. The rats surviving the experimental period were sacrificed by decapitation after 182 days. Hearts were perfused *in situ* with ice-cold heparinized physiological saline following cannulation of the proximal aorta and its ligation distal. The hearts were stopped in diastole by additional flushing with 7.45% potassium chloride. Transmural MI was clearly visible as a thin scar tissue with aneurysmatic bulging. Only rats with a transmural MI reaching from the base of the LV to its apex and including more than one-third of its circumference were included into further analysis. The resting pressure-volume curve analyses were performed in all the LVs, and the hearts were subsequently prepared either for chromatographical or histological determination of the collagen content.

### Resting pressure-volume curve

A pressure-volume-curve of the resting isolated LV was performed in all the probes. The tip of a double-lumen catheter (polyethylene tube, innerlumen 0.5 mm inside polyethylene tube, innerlumen 2.0 mm) was placed in the LV and ligated in the atrioventricular groove. The catheter was connected to a Statham pressure transducer to record a pressure-volume curve (infusion of 0.9% saline, 15 ml/h, ≤30 mmHg) (21). For each heart, two measurements were performed within 10 min after cardiac arrest. Pressure and volume were recorded every second. These data were used for an automated regression curve (best-fit curve) analysis. At pressure ranges of 0–3 mmHg, curves were generated following a linear model and were expressed as y = ax + b. The chamber stiffness is described by the slope of the curve, ‘a’. At pressure ranges of 3–10 and 10–30 mmHg, exponential curves had to be constructed as regression curves to fit the data. These curves can be described as y = cxd. Subsequent to calculating the logarithm, log(y) can be expressed as a linear function of ‘d’. In the exponential model the chamber stiffness is described by ‘d’. To determine the ventricular dilatation, the infused volumes at 5, 10 and 15 mmHg were measured for each heart.

### Collagen content

The collagen content in the non-infarcted area of the LV was assessed using two different methods. In 30 animals, the hydroxyproline content of the probes of the non-infarcted inferior left ventricular wall was measured by HPLC. Two probes, each weighing 100–150 mg, were taken from each heart. Hydroxyproline is solely found in collagen, constituting a fraction of 13.4%. Subsequent to homogenization in phosphate buffer and lyophilisation, the probes were hydrolyzed for 24 h at 114°C in 6 M HCl. Hydroxyproline bound to an ion-exchanger (Dowex W-X8, Sigma-Aldrich, St. Louis, MO, USA) in phosphate-citric-acid-buffer (pH 5.0), and was eluted from the ion-exchanger by heating up the probes to 115°C for 16 h and washing them with acetate-citrate-citric acid-buffer (pH 6.0). Aliquots were diluted in 20% acetate-buffer in methanol. The content of hydroxyproline in the probes was analyzed by HPLC with 7-chloro-4-nitrobenzofurazan as the fluorescent-labeling reagent. The results were expressed as nmol per probe and subsequently converted into microgram collagen per milligram dry weight of cardiac tissue.

The collagen content of the non-infarcted section of the LV was measured by planimetry of cryosections in 38 animals. The LVs were cut into four transversal cross-sectional slices of equal thickness and shock frozen in liquid nitrogen. Cryostat sections (7-μm) were cut from each probe and stained with Masson’s trichrome stain. Five optical fields (magnification, ×40) in the non-infarcted section of each cryostat section were analyzed. These five fields were dispersed across the slice circumferences, but no field with tissue defects due to the cutting process or further preparation, nor an optical field with large vessels respectively perivascular collagen were analyzed. The blue-stained collagen could be clearly distinguished from cardiac myocytes (with red-stained myofibrils and brown nuclei) or defects. This differentiation was performed automatically using an individualized macro for the NIH-Scion Image^®^ program (Scion Corp., Walkersville, MD, USA). The averaged gray scale values of all three colour channels were subtracted from the inverted gray scale values of the blue channel. A cut-off point was set to differentiate the blue staining. Calculation was performed as percentage of area using the pixel-based program. The hearts of these animals, which succumbed during the study period, were analyzed via histology.

### Infarct size and thickness of the scar

The infarct size was measured as the percentage of the inner and outer diameter in the cryostat sections of each of the four transversal cross-sectional slices (22). The thickness of the scar was measured three times in each of the cryostat sections.

### Statistics

All the values are expressed as mean ± standard error of the mean. Results were analyzed using a two-tailed Student’s t-test for unpaired data. For multiple comparisons analysis of variance was performed, followed by the Kruskal-Wallis test and two-sided Mann-Whitney U Test, where appropriate. Kaplan-Meier curves were constructed and statistical analysis of the survival curves was performed using the log-rank test. For histological analysis of the collagen content, scar thickness and infarct size, the raw data were aggregated for every animal and statistical analysis was performed as described above. P<0.05 was considered to indicate a statistically significant difference. PAWS Statistics for Windows, version 18 (SPSS Inc., Chicago, IL, USA) was used.

## Results

### Survival rates

**Figure 1:**
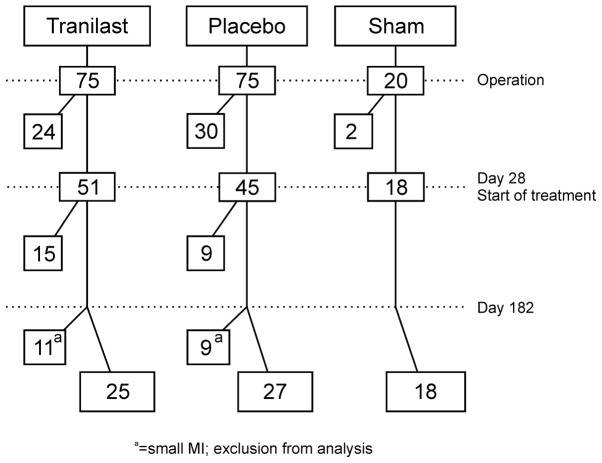
Flow diagram of the experimental settings. Branches to the left describe the mortality following the surgery and during the treatment period, and the exclusions due to small MI after day 182. MI, myocardial infarction.

The number of animals that succumbed during the different study periods and those included into the final analysis are shown in Fig. 1. The majority of the animals that succumbed up to day 28 had a large MI and signs of severe heart failure in the autopsies (pneumonia, n=2 and hematothorax, n=1). All the animals that succumbed within the treatment period of days 28–182 had a large MI, reaching from the base to the apex of the LV and comprising more than one-third of its circumference. No animal succumbed during the treatment period following the ShO.

**Figure 2:**
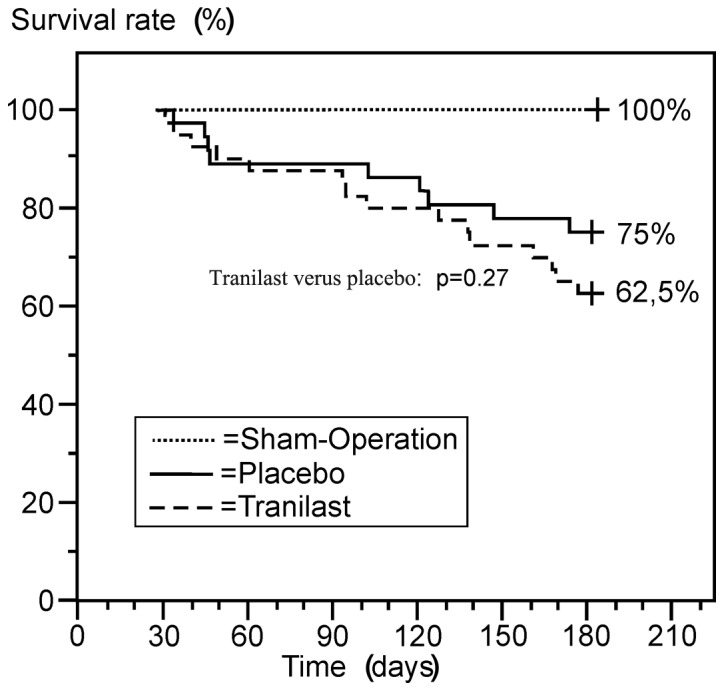
Kaplan-Meier analysis and survival rates six months after large myocardial infarction (tranilast, n=40; placebo, n=36; sham-operation, n=18).

At the end of the study period, 36 rats in the tranilast group, 36 rats in the placebo group and 18 rats following the ShO were sacrificed. Their organs and blood samples were obtained for analysis. There was no infarction of the right ventricle observed. None of the sham-operated rats had MI. In 11 rats of the tranilast group and in nine rats of the placebo group, the sizes of the MIs were too small to fit the aforementioned criteria. These animals were excluded from the Kaplan-Meier analysis and the weights of these lungs and hearts were analyzed separately. This led to a further reduction in the size of the groups, but enabled a comparison between the survivors and non-survivors. Finally, 40 animals that were treated with tranilast (15 succumbed and 25 survived) and 36 animals that were treated with placebo (nine succumbed and 27 survived) had a large MI, resulting in six-month survival rates of 62.5 and 75%, respectively. The curves in the Kaplan-Meier analysis were similar (Fig. 2).

The body weights at day 182 tended to differ among the three study groups, with the lowest body weight in the tranilast group, without reaching statistical significance (P=0.051). Induction of large MI led to a significant hypertrophy of the LV and right ventricle. However, the weights of the LV in the tranilast group were higher than those in the placebo group and this combination of lower body weight and higher weight of the LV led to a significant difference of the left-ventricular-index [heart weight (mg)/body weight (g)], between the two groups in the statistical analysis (Table II).

### Cardiac collagen content following large MI

**Figure 3:**
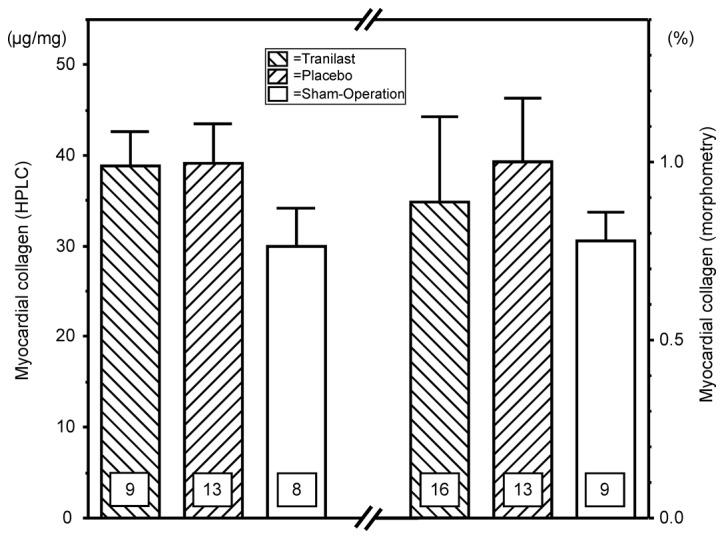
Intracardiac collagen content in the remote non-infarcted section of the left ventricle six months after large myocardial infarction. The collagen content was measured via HPLC or pixel-based analysis of stained cryosections on the left and right sides of the graph, respectively. Data are presented as the mean ± standard error of the mean. HPLC, high-performance liquid chromatography.

The cardiac hypertrophy following large MI was accompanied by an increase of intracardiac collagen content in the remote non-infarcted part of the LV. This aspect was shown in the histological analysis, as well as in the measurement of the hydroxyproline by HPLC. However, the two methods revealed that the difference compared with the hearts following the ShO was not statistically significant. The levels of collagen content were not significantly different between the treatment groups following MI (Fig. 3).

**Figure 4:**
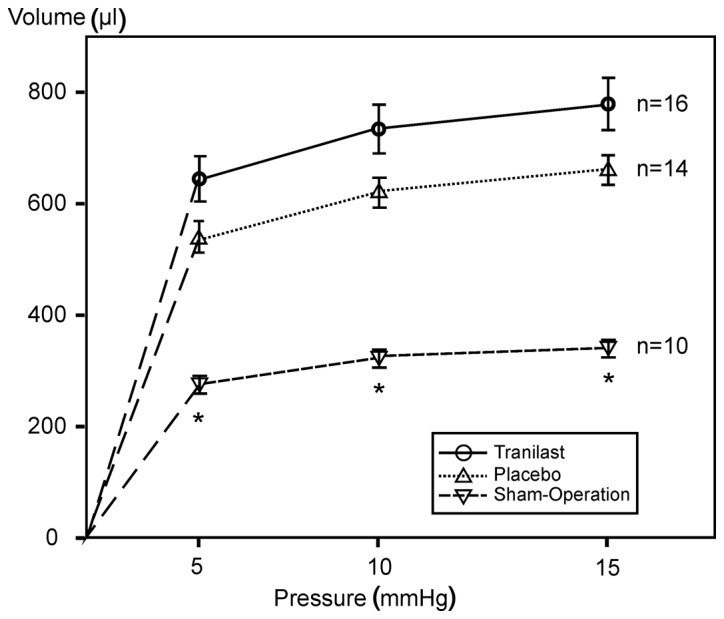
Pressure-volume association in the isolated left ventricle six months after large myocardial infarction or sham-operation. Data are presented as the mean ± standard error of the mean. ^*^P<0.001 vs. tranilast and placebo.

A severe dilatation of the LV following large MI could be found in the analysis of the pressure volume curves of the isolated LV (Fig. 4), as well as by measuring the circumference of the LV in the histological analysis. The infarct size was the same in the two treatment groups. Treatment with tranilast did not lead to a difference in the scar extension or thickness (Table III).

**Figure 5:**
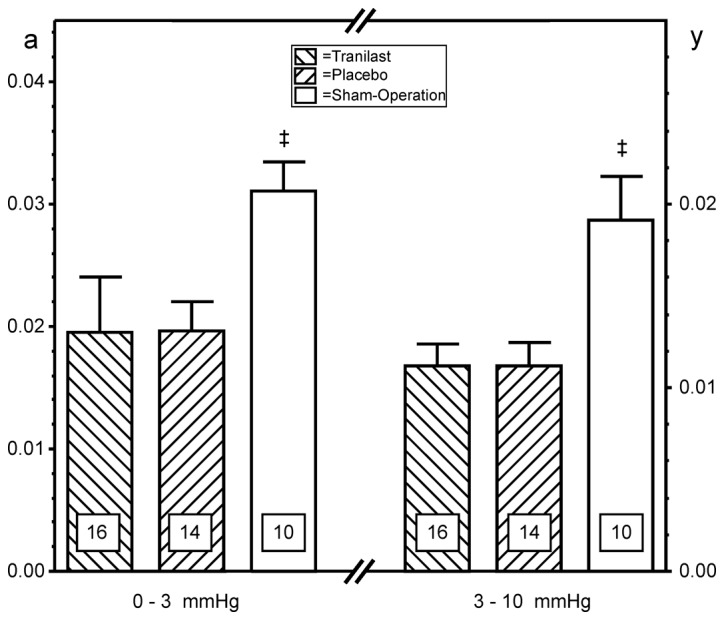
Calculation of the chamber stiffness variables. Analysis of resting pressure-volume curves of the isolated left ventricle, six months after large myocardial infarction or sham-operation. Left: ‘a’ derived by linear regression analysis (y = ax + b) at the pressure range, 0–3 mmHg. Right: ‘d’ of the isolated left ventricle derived by exponential regression analysis (y = c × xd) of resting pressure-volume curves at the pressure range, 3–10 mmHg. Data are presented as the mean ± standard error of the mean. ^‡^P<0.05 vs. tranilast and placebo.

Corresponding to the morphological changes following MI described above, a decrease of the chamber stiffness variables ‘a’ (pressure range, 0–3 mmHg) and ‘d’ (pressure range, 3–10 mmHg) was measured in the two treatment groups, without any significant difference between the treatment groups (Fig. 5).

### Non-survivors

**Figure 6:**
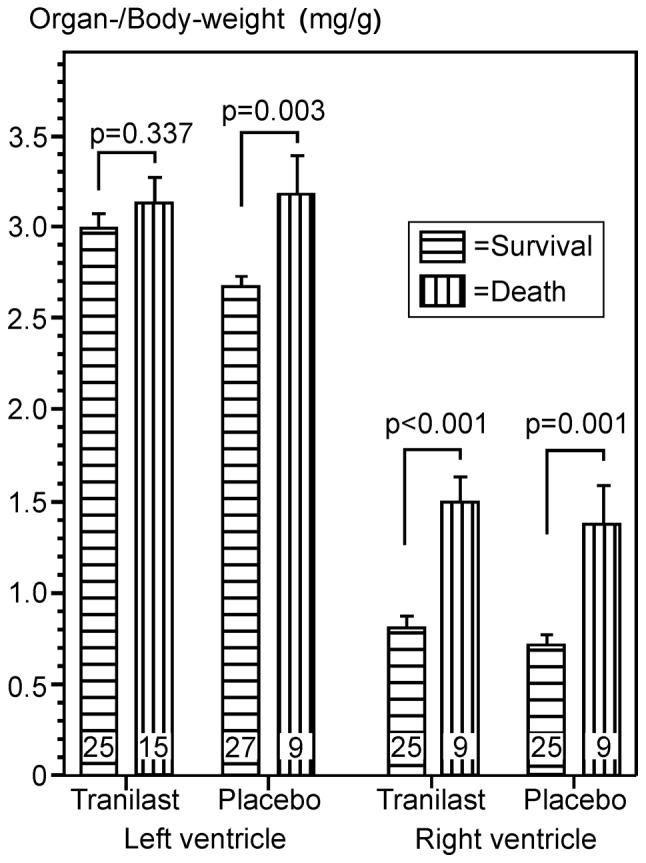
Weight of left and right ventricles per body weight in rats surviving the six month time period after large myocardial infarction, or succumbing in this time due to heart failure. Numbers in the bars show the group sizes (n). Data are presented as the mean ± standard error of the mean.

Following analysis of the organs from the animals that succumbed during the treatment period with large MI, no relevant difference between the treatment groups was found regarding the weight gain of the animals or the ventricular weight or the collagen content (histology) in the remote non-infarcted part of the LV (Table IV). Of note are the high weights of the ventricles compared with those of the animals that survived over the study period. This finding can be interpreted as a sign of an extensive cardiac hypertrophy, which led to a progressive ventricular failure and thereby to the early mortality of these animals (Fig. 6).

### Small MIs

Compared with the sham-operated hearts, the induction of small MIs led to a moderate increase of the weight, the intracardiac collagen content and a moderate dilatation of the LV, without reaching statistical significance. The ratios of the wet/dry weight of the lungs and the weights of the right ventricles were found at similar levels in all the groups. Chronic left ventricular failure induced following small MI was not severe enough to determine pulmonary edema and secondary right ventricular hypertrophy. Treatment with tranilast led to a lower weight gain compared with placebo. Regarding the other parameters, no statistically significant difference could be found between the treatment groups (Table V).

## Discussion

Treatment with the anti-inflammatory and antifibrotic drug tranilast, initiated four weeks after the induction of a large MI in the rat, did not attenuate the cardiac remodeling in chronic ischemic heart failure. There was no effect on the six-month mortality rate. These findings indicate that extremely late tranilast therapy does not positively influence recovery and remodeling in this setting.

By contrast, animal models of salt hypertensive rats, spontaneously hypertensive rats, renovascular hypertension and experimental diabetic cardiomyopathy have been reported to positively influence by tranilast therapy (13). Cardiac interstitial fibrosis and remodeling have been shown to improve, however, similarly the pathogenic mechanisms, as well as the therapeutic interventions, involve the entire LV in their whole circumference of the *per se* viable myocardium. When treatment is successful, the recovery process also comprises the entire circumference of the LV. The process of cardiac remodeling in the setting of definite myocardial infarction is different and can be described as a response of the remote non-infarcted section of the LV to cell damage in another area of the heart. These processes involve changes in ventricular geometry and shape during systolic contraction. The pathological background consists of myocyte hypertrophy, apoptosis and interstitial fibrosis in the remote non-infarcted area. Several studies examined the effects of revascularization procedures in acute myocardial ischemia. Therapeutic interventions, such as revascularization procedures, started in the early phase lead to the salvage of myocardial tissue at risk and reverse local dysfunction in the ischemic area. When effective, reverse geometric remodeling is observed and this is associated with beneficial effects on the left ventricular function, not only in the ischemic but also in the remote part of the LV (23). In addition, coronary revascularization of patients with viable, also known as hibernating, myocardium in the infarcted zone positively influenced cardiac remodeling and prevented further major cardiac events, but these interventions were not effective, if the ventricles were severely dilated and the ejection fraction was too low (24). In patients undergoing coronary revascularization during or subsequent to an acute MI, the left ventricular end-systolic volume could be identified as the most important discriminator for the development of heart failure and mortality in patients following MI, irrespective of the revascularization status (25). Any therapeutic approach other than revascularization during the acute phase of the MI may prevent further damage in the remote non-infarcted area, but the dilatation and change of geometry of the LV due to necrotic tissue cannot be reversed. In the present experimental setting, large MIs with prominent LV dilatation were induced. Starting treatment with tranilast four weeks after the induction of the large MI was too late to induce reverse cardiac remodeling. At this time the severe dilatation of the LV that was found after six months may already have been completed. Recently, it has been described that tranilast intervention from 24 h post-MI exacerbated infarct expansion, but delayed the commencement of treatment to seven days post-MI impeded LV remodeling (19). Taken together with the present data, starting on day 7 appears to be the optimal timing.

The extremely late time point of treatment initiation had been chosen to prevent an interaction of tranilast with the wound healing processes in the infarcted area of the LV with the risk of the formation of left ventricular aneurysms. Interactions of this type are described for other anti-inflammatory drugs administered during the acute phase of MI (26,27). During this maturation phase, the fibroblasts and vascular cells in the infarcted area undergo apoptosis. Cross-links are formed between the collagen bundles of the developing scar. After four weeks an organized assembly of collagen fibers in terms of scar tissue is found in the infarcted area of the LV (28). However, this appears not to be of relevance regarding the study endpoints.

Limitations of the present study include that it was performed in female Lewis rats. Compared with our previous results in male Lewis rats, lower levels of interstitial collagen were found measured in the two methods following ShO, as well as subsequent to the induction of a large MI (29). Similar results were reported for the animal model of uni-nephrectomized DOCA plus salt hypertensive mice. While the ratio of the heart weight to the body weight after four weeks was significantly increased in male animals, this was not the case in female animals (30). Gender differences can also be found concerning the mRNA levels for *TGF-β_1_* (31). The different results in male rats cannot be excluded. Care was taken during the procedure of the present study to produce large MIs by ligating the left anterior descending coronary artery as proximally as possible. Every animal that succumbed during the study period was autopsied to ensure that the mortality was caused by a large MI and subsequent heart failure. At the end of the study period, every heart was examined and only rats with comparable large MIs were included in the analysis. The aim was to create comparable study groups. The major drawbacks of this strategy were the high mortality rate in the perioperative time (44%) and an additional loss of animals at the end of the study period due to too small MIs (18%). There was only a small number of hearts available for the analysis of cardiac remodeling at the end of the study period.

Treatment with the anti-inflammatory and antifibrotic drug, tranilast, started four weeks after the induction of a large MI in the rat, does not attenuate or positively influence remodeling in chronic ischemic heart failure and survival rate. Further studies are required to explore the effects of tranilast on cardiac myocytes post-MI in more detail.

## Figures and Tables

**Table I:** Age and body weight of the rats at the time of randomization.

Characteristic	Plac (n=75)	Tra (n=75)	ShO (n=20)	P-value
Age, weeks	24.5±0.9	26.2±0.5	25.5±0.8	0.20
Body weight, g	219.8±2.5	215.1±3.1	223.8±3.6	0.26

Data are presented as the mean ± standard error of the mean. Groups: Plac, large myocardial infarction/placebo; Tra, large myocardial infarction/tranilast; ShO, sham-operation/placebo.

**Table II:** Body and organ weights six months after large myocardial infarction or sham-operation.

Parameter	Plac (n=27)	Tra (n=25)	ShO (n=18)
BW_182_, g	282.00±5.79	268.32±6.91	293.06±6.48
Weight gain, g^a^	62.21±7.60	51.15±6.10	67.07±5.73
LV, mg	742.2±12.3^b^	796.6±23.5^b^	614.1±14.2
LVI, mg/g	2.67±0.06^b^	2.99±0.09^b^,^d^	2.09±0.06
RV, mg	197.0±14.0^c^	209.0±15.8^b^	144.1±9.1
RVI, mg/g	0.70±0.05^c^	0.80±0.07^b^	0.49±0.03

Data are presented as the mean ± standard error of the mean.

a Weight gain is the difference of the body weight between day 182 and the time of surgery.

b P<0.001 vs. ShO,

c P<0.05 vs. ShO and

d P<0.05 vs. Plac.

Groups: Plac, large myocardial infarction/placebo; Tra, large myocardial infarction/tranilast; ShO, sham-operation/placebo. BW_182_, body weight at day 182; LV, Weight of the left ventricle; LVI, LV per body weight; RV, weight of the right ventricle; RVI, RV per body weight.

**Table III:** Histological evaluation of the infarct size, dilatation of the LV and scar thickness following large myocardial infarction or sham-operation (pixel-based analysis of stained cryosections).

Parameter	Tra (n=16)	Plac (n=13)	ShO (n=9)
Circumference LV, mm	26.67±0.85	28.32±0.96	14.77±0.64^a^
Scar length, mm	9.91±0.55	10.66±0.46	
Scar, % of circumference	37.29±1.80	40.44±1.67	
Scar thickness, mm	0.705±0.034	0.750±0.038	

Data are presented as the mean ± standard error of the mean.

a P<0.001 vs. Tra and Plac.

Groups: Plac, large myocardial infarction/placebo; Tra, large myocardial infarction/tranilast; ShO, sham-operation/placebo. LV, left ventricle.

**Table IV:** Body and organ weights and intracardiac collagen content in the remote non-infarcted part of the left ventricle obtained from the animals that succumbed during the treatment period following large myocardial infarction.

Parameter	Plac (n=9)	Tra (n=15)
Survival time, days	170±26	163±18
Collagen content, %^a^	0.98±0.13	1.00±0.44
BWR	1.15±0.13	1.11±0.05
LGR	5.72±0.37	5.37±0.24
LVI, mg/g	3.23±0.17	3.13±0.14
RVI, mg/g	1.36±0.21	1.48±0.15

Data are presented as the mean ± standard error of the mean.

a Collagen content is measured as the ratio of collagen-stained area over total stained area in the cryosections.

Groups: Plac, large myocardial infarction/placebo; Tra: large myocardial infarction/tranilast. BWR, ratio of body weight at time of mortality over that at the time of surgery; LGR, ratio of the wet over dry weight of the lungs; LVI, weight of left ventricle per body weight; RVI, weight of right ventricle per body weight.

**Table V:** Body and organ weights, collagen content and results of the pressure volume analysis of the isolated left ventricle, six months after small myocardial infarction or sham-operation.

Parameter	Plac (n=9)	Tra (n=11)	ShO (n=18)
BWR	1.44±0.03^a^	1.24±0.05	1.30±0.03
LGR	5.51±0.27	5.78±0.22	5.51±0.24
LVI, mg/g	2.31±0.21	2.34±0.16	2.09±0.06
RVI, mg/g	0.50±0.05	0.54±0.04	0.49±0.03
CSV at 0–3 mmHg	0.022±0.003	0.115±0.049	0.031±0.002
CSV at 3–10 mmHg	0.008±0.002	0.018±0.003	0.019±0.002
Volume 5 mmHg	398.2±43.2	326.7±40.7	283.4±16.2
Volume 10 mmHg	483.7±35.9	377.5±33.1	323.2±15.3
Volume 15 mmHg	546.3±33.8	411.6±28.1	342.7±15.6
Collagen HPLC (μg/mg)	41.31±4.83 (n=5)	39.98±5.87 (n=8)	29.98±4.18 (n=5)

a P<0.05 vs. Tra.

Data are presented as the mean ± standard error of the mean. Groups: Plac, large myocardial infarction/placebo; Tra, large myocardial infarction/tranilast; ShO, sham-operation/placebo. BWR, ratio of body weight at time of mortality over that at the time of surgery; LGR, ratio of the wet over dry weight of the lungs; LVI, weight of left ventricle per body weight; RVI, weight of right ventricle per body weight; CSV, chamber stiffness variable; HPLC, high-pressure liquid chromatography.
